# Characteristics of febrile urinary tract infections in older male adults

**DOI:** 10.1186/s12877-019-1360-3

**Published:** 2019-11-29

**Authors:** Alex Smithson, Javier Ramos, Esther Niño, Alex Culla, Ubaldo Pertierra, Michele Friscia, Maria Teresa Bastida

**Affiliations:** 1 Infectious Diseases Unit, Fundació Hospital de l’Esperit Sant, C/Avinguda Mossen Pons i Rabadà s/n, 08923 Santa Coloma Gramenet, Spain; 2 Internal Medicine Department, Fundació Hospital de l’Esperit Sant, C/Avinguda Mossen Pons i Rabadà s/n, 08923 Santa Coloma Gramenet, Spain; 3 Microbiology Laboratory, Fundació Hospital de l’Esperit Sant, C/Avinguda Mossen Pons i Rabadà s/n, 08923 Santa Coloma Gramenet, Spain

**Keywords:** Older adults, Males, Febrile urinary tract infection

## Abstract

**Background:**

Urinary tract infections (UTI) are among the most frequent bacterial infections in older adults. The aim of the study was to analyse the existence of differences in clinical features, microbiological data and risk of infection by multidrug-resistant organisms (MDRO) between older and non-older men with febrile UTI (FUTI).

**Methods:**

This was an ambispective observational study involving older males with a FUTI attended in the Emergency Department. Variables collected included age, comorbidity, diagnostic of healthcare-associated (HCA)-FUTI, clinical manifestations, hospitalization, mortality, and microbiological data.

**Results:**

Five hundred fifty-two males with a FUTI, 329 (59.6%) of whom were older adults, were included. Older males had a higher frequency of HCA-FUTI (*p* <  0.001), increased Charlson scores (*p* <  0.001), had received previous antimicrobial treatment more frequently (*p* <  0.001) and had less lower urinary tract symptoms (*p* <  0.001). Older patients showed a lower frequency of FUTI caused by *E. coli* (*p* <  0.001) and a higher rate of those due to *Enterobacter spp.* (*p* = 0.003) and *P. aeruginosa* (*p* = 0.033). Resistance rates to cefuroxime (*p* = 0.038), gentamicin (*p* = 0.043), and fluoroquinolones (*p* <  0.001*)* in *E. coli* isolates and the prevalence of extended-spectrum beta-lactamase and AmpC producing *E. coli* and *Klebsiella spp.* strains (*p* = 0.041) and MDRO (*p* <  0.001) were increased in older males. Inadequate empirical antimicrobial treatment (*p* = 0.004), frequency of hospitalization (*p* <  0.001), and all cause in-hospital mortality (*p* = 0.007) were higher among older patients. In the multivariate analysis, being admitted from an long term care facility (OR 2.4; 95% CI: 1.06–5.9), having a urinary tract abnormality (OR 2.2; 95% CI: 1.2–3.8) and previous antimicrobial treatment (OR 3.2; 95% CI: 1.9–5.4) were associated to FUTI caused by MDRO.

**Conclusions:**

Older male adults with a FUTI have different clinical characteristics, present specific microbiological features, and antimicrobial resistance rates. In the multivariate analysis being an older male was not associated with an increased risk of FUTI caused by MDRO.

## Background

The number of older adults is increasing in virtually every country and will continue to grow over the coming decades. According to the 2018 Ageing Report, by 2050 older adults will represent 28% of the European Union citizens with an old-age dependency ratio of 55% of the working-age population [1]. With an ageing and highly dependent society the burden of infectious diseases is expected to increase mainly due to a high prevalence of older people with comorbid conditions, long-term institutionalization, frailty and a weakened immune system [2]. Infections in the older adults have specific characteristics that have to be recognised in order to provide an optimal care such as uncommon clinical presentations, high rates of organ failure and antimicrobial interactions together with an increased severity and risk of infections caused by multidrug-resistant organisms (MDRO) [2].

Urinary tract infections (UTI) are one of the most common bacterial infections affecting geriatric patients and the main source of community acquired bacteremia [3, 4]. After pneumonia, UTI represent the second cause of hospital admission due to an infection in the older adults [5]. Despite their high frequency, UTI are frequently overdiagnosed due to the high prevalence of asymptomatic bacteriuria (ASB) in older populations which may lead to an incorrect diagnostic and unnecessary antimicrobial treatment in the presence of non-specific urinary symptoms [3, 6]. This increased exposure to antimicrobials is one of the main factors explaining the high prevalence of UTI due to MDRO in the older adults including those caused by extended-spectrum betalactamase (ESBL) producing Enterobacteriaceae [7, 8].

Women are more prone to experience UTI although rates tend to equalise in ageing males due to impaired normal voiding mainly associated with benign prostatic hyperplasia (BPH) [9]. The main consequence of these voiding difficulties is the generation of a retrograde turbulent urine flow, enabling the ascension of uropathogens to the bladder and eventually into the prostate, which explains the high frequency of prostatic involvement in males with UTI [10]. Therefore, most febrile UTI (FUTI) in males should be considered as acute bacterial prostatitis (ABP) and treated using antimicrobials with an adequate prostatic diffusion such as fluoroquinolones (FQ) [11]. Increasing antimicrobial resistance rates hinder the eradication of the bacteria from the prostate thus becoming the source of recurrent UTI in males [3].

The majority of the studies that have analysed the characteristics of FUTI in older populations have focused on women [12, 13]. Therefore, we believe there is a knowledge gap regarding the characteristics of FUTI in older males. The objective of this study was to investigate the existence of differences in clinical features, microbiological data and risk of infection by MDRO between older and non-older men with FUTI.

## Methods

### Study design

This was an ambispective observational study in which we evaluated males aged > 18 years with a FUTI and a positive urine culture seen at the emergency department (ED) of a 165-bed primary care hospital from 2008 to 2015. Information was recorded retrospectively until 2009 and prospectively thereafter. FUTI was defined as an axillary temperature > 38 °C, measured at home or the ED**,** and one or more lower urinary tract symptoms (LUTS) including urinary urgency, frequency and/or dysuria. In the absence of LUTS the patient was diagnosed as having a FUTI when no additional focus of infection was identified. FUTI episodes with negative or polymicrobial urine cultures were excluded from the analysis. The study was approved by the Clinical Research Ethics Committee of the “La Fundació Unió Catalana d’Hospitals” (approval number: CEIC 16/58). Written informed consent was obtained from patients prospectively included.

### Study population, description of measures and definitions

Patients were divided into older and non-older males. Male patients aged 65 years or above were identified as older patients. Males aged between 65 and 79 were considered early older patients while those aged 80 years or above were considered to be late older patients. Men under 65 were defined as non-older. The variables collected from each patient included age, dementia, healthcare-associated (HCA)-FUTI, diabetes mellitus, kidney failure, cirrhosis, neoplastic disease, chronic obstructive lung disease, heart failure, use of immunosuppressives in the previous 30 days, comorbidity Charlson age adjusted score [14], antibiotic treatment in the prior 30 days, existence of urinary tract abnormalities or past UTI episodes. We also recorded information regarding the patients symptoms and from their physical examination at the ED including the axillary temperature and the mean arterial pressure (MAP). Patients were tracked until hospital discharge or death**.** In case of recurrent FUTI, only the initial episode was analysed.

We considered that the patient had a HCA-FUTI in case of: hospitalization for 2 or more days in the previous 90 days; residence in an long-term care facility (LTCF); intravenous therapy at home or in a day hospital, hemodialysis, specialized nursing care, invasive urinary tract instrumentation, 30 days before the FUTI; presence of an indwelling urethral catheter (IUC) in place or removed 48 h before the infection including short term (< 30 days) and long term (> 30 days) catheters. Patients without any of the prior criteria were catalogued as having a community-acquired FUTI.

Urine specimens were collected from clean-catch midstream urine. In case of long-term IUC it was recommended to obtain urine samples from a newly inserted catheter. Samples were cultured on CLED and MacConkey agar and considered positive in case of bacterial growth > 10^3^ colony-forming units/mL. Standard biochemical methods were used for microbiological identification. Antimicrobial susceptibility of the isolated bacteria was performed by the disk diffusion technique according to the recommendations of the Clinical and Laboratory Standards Institute. The antibiotics tested included: amoxicillin, amoxicillin-clavulanate acid, cefuroxime, ceftriaxone, imipenem, gentamicin, nalidixic acid, ciprofloxacin, co-trimoxazole, and fosfomycin. Resistant and intermediate strains were pooled together for statistical analysis. The isolates were screened for ESBLs and Amp-C β-lactamases production by phenotypic methods. Microorganisms were considered as MDRO in case of expressing an acquired resistance to at least one agent in three or more antimicrobial categories [15].

The 2001 definitions were used to define severe sepsis and septic shock [16]. Inadequate empirical antimicrobial treatment (IEAT) was defined in case the isolated uropathogen was resistant to the first antibiotic administered at the ED or when treating with a third-generation cephalosporin (TGC) in monotherapy FUTI due to microorganisms with inducible chromosomal beta-lactamases (mainly *Enterobacter spp., Serratia spp., C. freundii, M. morgagnii* and *Providencia spp*). All-cause in-hospital mortality was defined when the patient died during the hospitalization independently of its causes while FUTI-related in-hospital mortality when the patient died due to the infection.

### Statistical analysis

Dichotomous variables are presented as number of patients (percentages) and continuous variables as mean (+ standard deviations). Dichotomous variables were compared by using the Chi-square or the Fisher exact test as appropriate while continuous variables were compared by the Student *t-*test or the Mann-Whitney U-test. A logistic regression analysis was performed to identify independent variables associated with infections caused by MDRO. Variables with a *p* <  0.1 in the univariate analysis were included in the multivariate analysis. Model validity was evaluated by the Hosmer-Lemeshow test for estimating goodness of fit. Statistical significance was defined as a two-tailed *P* value of < 0.05. Data were analyzed with SPSS 20.0.

## Results

Five hundred and fifty-two males with a FUTI attended in the ED were included in the study. Three hundred and twenty-nine (59.6%) were older and 223 (40.4%) non-older patients. Among the older males, 210 (63.8%) were classified as early older and 119 (36.2%) as late older patients. Overall, 356 patients (64.5%) had one or more chronic medical conditions. Of the included patients, 239 (43.4%) had a previous diagnosis of BPH which represented the most common urinary tract abnormality. The baseline characteristics, clinical features, empirical treatment and outcomes of the patients are shown in Table 1. Older males had an increased frequency of HCA-FUTI, and the vast majority of the medical conditions analysed and thus had higher Charlson scores. Previous UTI episodes, the existence of urinary tract abnormalities, and antimicrobial intake in the preceding month, mainly with FQ, were also more common in older patients. In addition, older males more frequently had an IUC half of which were long-term catheters and the rest short-term catheters mainly placed after urological surgery. None of the patients were on hemodialysis.

**Table 1:** Basal characteristic, clinical features, empirical antimicrobial treatment and outcomes of included patients and analysis of existing differences between older and non-older patients with a febrile urinary tract infection

Characteristics^a^	All patients (*n* = 552)	Older (*n* = 329)	Non-older (*n* = 223)	*P* value
Baseline conditions				
Age (years)	66.1 + 16.7	77.3 + 7.6	49.5 + 12.2	< 0.001
HCA-FUTI	203 (36.8)	177 (53.8)	26 (11.7)s	< 0.001
Previous hospital admission	130 (23.6)	114 (34.7)	16 (7.2)	< 0.001
Long term care facility	46 (8.3)	43 (13.1)	3 (1.3)	< 0.001
IV home-day hospital treatment	13 (2.4)	9 (2.7)	4 (1.8)	0.47
Specialized nursing care	7 (1.3)	6 (1.8)	1 (0.4)	0.25
Indwelling urinary catheter	76 (13.8)	66 (20.1)	10 (4.5)	< 0.001
Long-term IUC	40 (7.2)	33 (10)	7 (3.1)	0.002
Short- term IUC	36 (6.5)	33 (10)	3 (1.3)	< 0.001
Invasive urinary intrumentation	110 (19.9)	98 (29.8)	12 (5.4)	< 0.001
Dementia	64 (11.6)	62 (18.8)	2 (0.9)	< 0.001
Diabetes mellitus	148 (26.8)	113 (34.3)	35 (15.7)	< 0.001
Chronic kidney failure	54 (9.8)	49 (14.9)	5 (2.2)	< 0.001
Cirrhosis	9 (1.6)	5 (1.5)	4 (1.8)	1
Neoplasia	81 (14.7)	72 (21.9)	9 (4)	< 0.001
Heart failure	8 (1.4)	7 (2.1)	1 (0.4)	0.15
Chronic obstructive lung disease	100 (18.1)	90 (27.4)	10 (4.5)	< 0.01
Immunosuppressive or corticosteroid treatment	11 (2)	7 (2.1)	4 (1.8)	1
Charlson score	3.6 + 2.5	5.1 + 1.7	1.3 + 1.6	< 0.001
Previous UTI	222 (45.5)	152 (53.7)	70 (34.1)	< 0.001
Urinary tract abnormality	339 (61.5)	252 (76.8)	87 (39)	< 0.001
BPH	239 (43.4)	187 (57)	52 (23.3)	< 0.001
Urogenital cancer	48 (8.8)	44 (13.6)	4 (1.8)	< 0.001
Previous antibiotic treatment	179 (32.6)	135 (41.3)	44 (19.8)	< 0.001
Aminopenicillins	60 (11)	42 (13)	18 (8.2)	0.081
Fluoroquinolones	54 (9.9)	41 (12.7)	13 (5.9)	0.010
TGC	16 (2.9)	11 (3.4)	5 (2.3)	0.44
Fosfomycine	10 (1.8)	9 (2.8)	1 (0.5)	0.055
Clinical presentation				
LUTS	359 (65.3)	172 (52.4)	187 (84.2)	< 0.001
Flank pain	89 (16.2)	31 (9.5)	58 (26.1)	< 0.001
Physical examination				
Temperature	37.7 + 1	37.7 + 1.1	37.7 + 0.9	0.85
MAP (mmHg)	93.4 + 15.3	91.1 + 15.3	96.9 + 14.7	< 0.001
Lumbar tenderness	94 (22.1)	40 (17.3)	54 (27.7)	0.010
Prostatic tenderness	81 (42.6)	35 (37.2)	46 (47.9)	0.13
Laboratory				
WBC count (leukocytes/mm^3^)	13,903 + 5743	13,348 + 5536	14,759 + 5961	0.006
Creatinine (mg/dl)	1.2 + 0.7	1.4 + 0.8	1.1 + 0.4	< 0.010
Bacteremia	120 (31.6)	92 (38)	28 (20.3)	< 0.010
Treatment				
Empirical treatment with TGC	307 (55.6)	176 (53.5)	131 (58.7)	0.22
Empirical treatment with carbapenems or PT	59 (10.7)	46 (14)	13 (5.8)	0.002
IEAT	95 (17.2)	69 (21)	26 (11.7)	0.004
OPAT	71 (12.9)	54 (16.4)	17 (7.6)	0.002
Outcomes				
Severe sepsis or septic shock	17 (3.1)	12 (3.6)	5 (2.2)	0.35
Hospital admission	266 (48.2)	197 (59.9)	69 (30.9)	< 0.001
Length of hospitalisation (days)	2.6 + 4.1	3.3 + 4.6	1.4 + 2.9	< 0.001
Length antimicrobial therapy (days)	16.3 + 6.7	16 + 7	16.8 + 6.5	0.19
All-cause in-hospital mortality	15 (2.7)	14 (4.3)	1 (0.4)	0.007
Attributable in-hospital mortality	8 (1.4)	8 (2.4)	0	0.024

*Abbreviations*: *BPH* Benign prostatic hyperplasia, *HCA-FUTI* Healthcare-associated febrile urinary tract infection, *IEAT* Inadequate empirical antimicrobial treatment, *IUC* Indwelling urinary catheter, *LUTS* Lower urinary tract symptoms, *MAP* Mean arterial pressure, *OPAT* Outpatient parenteral antimicrobial therapy, *PT* Piperacillin-tazobactam, *TGC* Third-generation cephalosporins, *UTI* Urinary tract infections, *WBC* White blood cell

^a^Data presented as mean + standard deviation (SD) or numbers of patients (percentages)

Regarding the clinical presentation, older males presented less LUTS, flank pain and lumbar tenderness with a similar degree of fever than non-older males. The MAP was lower in the older patients although no differences in the frequency of severe sepsis and septic shock were found when compared to non-older patients. Besides, older males presented an increased frequency of bacteremic FUTI.

The isolated uropathogens and their antimicrobial resistant pattern is shown in Table 2. *E. coli* was the predominant pathogen in older and non-older patients followed by *K. pneumonia* and *P. aeruginosa*. *E. coli* was less commonly isolated in older males, although it was still the most common causal uropathogen. Resistance rates to cefuroxime, gentamicin, and FQ were higher in *E. coli* strains isolated from older patients. When evaluated together, ESBL and AmpC producing *E. coli and Klebsiella spp.* were more prevalent among older patients. No carbapenemase-producing *Enterobacteriaceae* strains were isolated during the study period. The frequency of *Enterobacter spp.* and *P. aeruginosa* was also higher in older males. Patients with long-term IUC had a lower frequency of FUTI caused by *E. coli* (35% vs 74%; *p* <  0.001), and an increased risk of those caused by *K. pneumoniae* (22.5% vs 7.4%; *p* 0.004), *P. aeruginosa* (17.5% vs 3.5%; *p* 0.001), and *P. mirabilis* (10% vs 2.5%; *p* 0.028) together with higher rates of infections caused by MDRO (Table 3) when compared to patients with short-term IUC.

**Table 2:** Isolated uropathogens and antimicrobial resistance profiles

Uropathogens^a^	All patients (*n* = 552)	Older (*n* = 329)	Non-Older (*n* = 223)	*P* value
*Escherichia coli*	393 (71.2)	206 (62.6)	187(83.9)	< 0.001
Amoxicillin	254 (69.2)	137 (71.4)	117 (67)	0.35
Amoxicillin-clavulanate	52 (13.3)	33 (16.2)	19 (10.2)	0.080
Cefuroxime sodium	38 (9.7)	26 (12.6)	12 (6.4)	0.038
Ceftriaxone^b^	35 (8.9)	23 (11.2)	12 (6.4)	0.099
Gentamicin	30 (7.7)	21 (10.2)	9 (4.8)	0.043
Fluoroquinolones	153 (38.9)	100 (48.5)	53 (28.3)	< 0.001
Co-trimoxazol	102 (26.8)	56 (28.3)	46 (25.3)	0.51
Fosfomycine	8 (2.1)	6 (3.1)	2 (1.1)	0.28
*Klebsiella spp.*^c^	56 (10.1)	40 (12.2)	16 (7.2)	0.057
Amoxicilline-clavulanate	13 (23.2)	8 (20)	5 (31.3)	0.48
Ceftriaxone^d^	12 (21.4)	9 (22.5)	3 (18.8)	1
Fluoroquinolones	15 (26.8)	12 (30)	3 (18.8)	0.51
Co-trimoxazol	11 (20.8)	8 (21.1)	3 (20)	1
ESBL and AmpC producing *E. coli* and *Klebsiella* spp.	48 (10.7)	33 (13.4)	15 (7.4)	0.041
*Enterobacter spp.*^e^	21 (3.8)	19 (5.8)	2 (0.9)	0.003
*Serratia spp.*	4 (0.7)	3 (0.9)	1 (0.4)	0.65
*Citrobacter spp.*	4 (0.7)	1 (0.3)	3 (1.3)	0.3
*Morganella spp.*	5 (0.9)	4 (1.2)	1 (0.4)	0.65
*Proteus mirabilis*	17 (3.1)	14 (4.3)	3 (1.3)	0.052
*Pseudomona aeruginosa*	25 (4.5)	20 (6.1)	5 (2.2)	0.033
Fluoroquinolones	7 (28)	7 (35)	0	0.27
Other Gram negative bacteria^f^	2 (0.4)	2 (0.6)	0	0.51
Gram positive bacteria	22 (4)	17 (5.2)	5 (2.2)	0.085
*Enterococcus spp.*^g^	16 (2.9)	12 (3.6)	4 (1.8)	0.20
*Staphylococcus spp.* ^h^	6 (1.1)	5 (1.5)	1 (0.4)	0.41
*Candida spp.*^i^	3 (0.5)	3 (0.9)	0	0.27

*Abbreviations*: *ESBL* extended-spectrum beta-lactamase

^a^Data presented as number of patients (percentages). ^b^Include 32 (91.4%) ESBL and 3 (8.6%) AmpC producing strains. ^c^Include 47 (84%) *K. pneumoniae* and 9 (16%) *K. oxytoca* strains. ^d^Include 9 (75%) ESBL and 3 (25%) AmpC producing strains. ^e^Include 18 (85.7%) *E. cloacae* and 3 (14.3%) de *E. aerogenes strains.* ^f^Include one *P. stuartii* strain and one *A. baumannii* strain. ^g^Include 11 (68.7%) *E. faecalis*, 4 (25%) *Enterococcus spp.* and 1 (6.3%) *E. faecium* strains. ^h^Include 6 (83.3%) *S. aureus* and 1 (16.7%) *S. capitis* strain. ^i^Include 2 *C. albicans* and 1 *C. tropicalis* strains

**Table 3:** Univariate and multivariate analysis of risk factors associated with febrile urinary tract infection caused by multidrug-resistant organisms

Characteristics^a^	MDRO infection (*n* = 135)	Non-MDRO infection (*n* = 414)	*P* value^2^	OR (95% CI)	*P* value^3^
Older males	98 (72.6)	228 (55.1)	< 0.001		
Previous hospital admission	46 (34.1)	81 (19.6)	0.001		
Long term care facility	23 (17)	22 (5.3)	< 0.001	2.4 (1.06–5.9)	0.037
IV home-day hospital treatment	1 (0.7)	12 (2.9)	0.2		
Specialized nursing care	2 (1.5)	5 (1.2)	0.68		
Indwelling urethral catheter	29 (21.5)	46 (11.1)	0.002		
Long-term IUC	21 (15.6)	19 (4.6)	< 0.001		
Short-term IUC	8 (5.9)	27 (6.5)	0.8		
Invasive urinary Instrumentation	38 (28.1)	69 (16.7)	0.003		
Dementia	27 (20)	36 (8.7)	< 0.001		
Diabetes mellitus	40 (29.6)	106 (25.6)	0.36		
Chronic kidney failure	16 (11.9)	37 (8.9)	0.32		
Cirrhosis	2 (1.5)	7 (1.7)	1		
Neoplasia	22 (16.3)	57 (13.8)	0.46		
Heart failure	4 (3)	4 (1)	0.1		
Chronic obstructive lung disease	30 (22.2)	70 (16.9)	0.16		
Immunosuppressive or corticosteroid treatment	1 (0.7)	10 (2.4)	0.31		
Previous UTI	74 (62.2)	148 (40.1)	< 0.001		
Urinary tract abnormality	100 (74.6)	236 (57)	< 0.001	2.2 (1.2–3.8)	0.006
Previous antibiotic treatment	71 (53)	107 (26)	< 0.001	3.2 (1.9–5.4)	< 0.001

*Abbreviations*: *CI* Confidence interval, *IUC* Indwelling urinary catheter, *MDRO* Multidrug-resistant organisms, *OR* Odds ratio, *UTI* Urinary tract infections

^a^Data presented as numbers of patients (percentages).^2^Univariate analysis.^3^Multivariate analysis

As shown in Table 3 the frequency of infections caused by MDRO was higher in older patients. In the multivariate analysis independent risk factors for having a FUTI caused by a MDRO were coming from an LTCF, having a urinary tract abnormality and recent antimicrobial treatment but not with being an older male patient.

Regarding empirical antimicrobial treatment, TGC was the main therapeutic option used in older and non-older patients. A higher number of older males received empirical treatment with either a carbapenem or with piperacillin-tazobactam (Table 1). Older patients more frequently received an IEAT and were treated with OPAT, mainly with ertapenem in 25 (46.3%) cases and with ceftriaxone in 23 (42.6%) patients. Older males also required hospital admission more often with longer length of hospitalization and exhibited an increased crude and attributable in-hospital mortality compared with non-older patients (Table 1). A multivariate analysis of risk factors associated to in-hospital mortality was not performed due to the small number of deaths among the included patients.

## Discussion

According to our study, older males with a FUTI present specific clinical characteristics, different microbiological and antimicrobial-resistant patterns and outcomes when compared to non-older males. Due to the high frequency of UTI among older patients it is important to consider these differences when attending geriatric males with FUTI in the ED in order to provide an optimal therapeutic care.

Normal voiding is the most important defense against UTI. Increased postvoid residual volume, which occurs in ageing males mainly related to BPH, causes urinary stasis and predisposes to UTI. Urinary tract abnormalities and IUC are additional contributors to UTI in geriatric patients [3, 17, 18]. As expected, a higher frequency of urinary tract abnormalities, mainly BPH but also urogenital cancer, was observed in older patients. In a study by Søgaard et al., acute pyelonephritis in patients older than 50 years was identified as a marker of urogenital cancer suggesting that older patients with FUTI should be evaluated to rule out this possibility [19].

Regarding the clinical presentation, more than half of the included older males presented LUTS, in accordance to what has been previously described [3]. Despite the frequent existence of urinary symptoms in older males, we observed a lower frequency of LUTS, flank pain and lumbar tenderness compared to non-older patients. A lower frequency of localizing urinary symptoms in older patients with a FUTI has been observed in previous studies, particularly in those living in LTCF [3, 12, 20]. Although fever is frequently absent or blunted in geriatric patients with an acute infection, no differences in the degree of fever were seen between older and non-older patients [21]. Fever without localizing genitourinary signs and symptoms is the most common presentation of symptomatic UTI in residents from an LTCF with an IUC [3]. As pyuria and bacteriuria are practically constant in patients with a long-term IUC when fever is present an alternative focus of infection has to be ruled out before being attributed to a urinary infection [18].

The evaluation of UTI in geriatric patients can be challenging due to defective communication, functional disability and the existence of chronic LUTS usually not attributable to UTI [3, 18]. Although non-specific changes in the clinical status of older patients such as delirium, increased falls, fatigue or loss of appetite, are frequently attributed to UTI, particularly in the presence of a positive urine culture, the value of these symptoms as indicators of a proper UTI has been questioned [3, 6, 22, 23]. According to a recent decision tool for deciding empiric antimicrobial treatment in frail older adults with suspected UTI and the 2019 IDSA guidelines for the management of ASB, most non-specific symptoms in the absence of LUTS or other signs of infection should not be attributed to UTI [24, 25]. Therefore, due to the high prevalence of pyuria and ASB in older patients, urinalysis should only be performed when signs and symptoms of UTI are present [3, 18, 24].

In our study, FUTI in older males were less commonly caused by *E. coli* with an increased frequency of infections due to other gram-negative bacteria, which is in accordance with previous studies [2]. *E. coli* isolated from older males exhibited increased resistance rates to cefuroxime, gentamicin, and FQ. We also observed a higher prevalence of ESBL and AmpC producing *E. coli* and *Klebsiella spp.* strains with rates similar to the ones reported by Alvarez et al. among older patients with UTI [26]. The impact of age in the frequency of FUTI caused by MDRO has been observed in some studies [8, 12] but not in others [27, 28]. Interestingly, men seem to have an increased risk of infection with ESBL-producing Enterobacteria and other MDRO compared to women [8, 28, 29].

In the multivariate analysis, coming from an LTCF, having a urinary tract abnormality and recent antimicrobial use were independently associated to FUTI caused by MDRO. Residing in an LTCF has been identified as a risk factor for infection caused by ESBL-producing Enterobacteria [8, 28, 29]. Pulcini et al. showed that older people from nursing homes had a 40% risk of having an antibiotic-resistant Enterobacteriaceae isolated from the urine compared to their community peers [30]. More recently, McKinnell et al. estimated a prevalence of MDRO colonization in older people from nursing homes and long-term acute care facilities of 65 and 80% respectively [31]. All these studies support the role of LTCF as reservoirs of MDRO.

Recent exposure to antimicrobials, as in previous studies, was also associated with FUTI due to MDRO [8, 28]. Antimicrobial consumption is particularly high among residents of LTCF. It has been estimated that 75% of the residents of LTCF who stay for 6 months or longer will receive at least one course of antibiotics, commonly for suspected UTI in older patients with pyuria or ASB and non-specific urinary symptoms [18]. Given the burden of inappropriate management of ASB, particularly in LTCF, it is crucial to establish antimicrobial stewardship programs that have demonstrated to decrease antimicrobial consumption [32].

In an attempt to decrease antimicrobial overtreatment it has been recommended to avoid therapy in older patients with ASB and non-specific urinary symptoms or even to defer it in case of mild urinary symptoms awaiting urine culture results [3]. These recommendations have been questioned in an article by Gharbi et al. in which the authors suggest that not prescribing or deferring antimicrobials in older patients with a UTI increases the risk of bloodstream infection, hospital admission and all-cause mortality [33].

Older males with underlying urinary tract abnormalities also had an increased risk of infection by MDRO. Although having urinary abnormalities has been considered a risk factor for having UTI caused by resistant uropathogens [34] different studies show conflicting results. While Lee et al. observed a relationship between bacteremic UTI caused by MDRO and different urinary tract abnormalities [35], in a study by Gomila et al. only the presence of an IUC was associated with UTI caused by MDRO in hospitalised patients [28].

Once a FUTI is diagnosed, an empirical antimicrobial treatment has to be started to avoid progression to sepsis or even septic shock. In our study, older males received more frequently a carbapenem or piperacillin-tazobactam than non-older male. The decision to indicate a broad spectrum antibiotic should be based on the severity of the infection, the presence of known risk factors for MDRO and the local antimicrobial resistance rates. An increase in the consumption of carbapenems has been identified as a driver for carbapenemase resistance among Enterobacteriaceae strains [36]. In the present study, 21% of the older males received an IAET, which was lower than the frequency described in previous studies in older patients with UTI with rates that ranged from 27.4 to 29.3% [26, 37, 38].

Regarding directed antimicrobial therapy, as prostatic involvement in males with FUTI is common, FQ have been considered the first therapeutic choice due to their high degree of prostatic penetration [11]. The use of FQ is limited due to high resistant rates and the risk of severe adverse events related to its use, including aortic aneurysms [39]. Co-trimoxazol is an alternative but again high resistance rates and the risk of acute kidney injury and hyperkalemia in older adults hampers its use [40]. Fosfomycine has emerged as an additional option for directed therapy as it achieves reasonable intraprostatic concentrations and it is active against most MDRO including ESBL strains [41]. Regarding the duration of antimicrobial treatment in males with FUTI, very few studies have analysed this topic. Ulleryd et al. showed that 14 day was not inferior to 28 day treatment while more recently van Niewkoop et al. demonstrated that 7 days was less efective than 14 day treatment in males with FUTI [42, 43]. In our study, older and non-older patients received slightly more than 2 weeks of antimicrobial therapy.

Management of IUC in FUTI is also important, particularly in older patients who are frequently catheterized. IDSA guidelines recommend replacing a long-term IUC to hasten the resolution of symptoms in catheter-associated UTI [44]. This recommendation has recently been questioned in a study by Babich et al. in which the authors found no clinical benefit from replacing a long-term IUC at the onset of a catheter-associated UTI [45]. In a later editorial letter, the authors were questioned about the IDSA guidance to obtain a urine culture from a freshly placed catheter as a sample from a long-term IUC might not reflect the status of the infection in the bladder. The authors replied that in 97% of cases of bacteremia the same species was isolated from the urine while recognizing the need for further research with randomized controlled trials [46].

As in previous studies, older patients required hospital admission more frequently with longer length of hospitalization [3, 12]. Also, older patients had an in-hospital mortality rate of 4.3%, which was higher compared to non-older patients but significantly lower than the rates reported in previous studies in geriatric patients with UTI with rates that ranged from 8.9 to 33% [26, 37, 47, 48]. Severe sepsis and septic shock are the risk factors that most robustly have been associated with increased mortality in patients with FUTI [26, 38, 47]. Despite several studies have found age to increase mortality in patients with sepsis [26, 38, 47], others point out that preexisting medical conditions, including chronic kidney failure, could play a more prominent role [49, 50].

The present study has some potential limitations that have to be taken into consideration. Firstly, the study had a retrospective component and was performed in a single hospital. Secondly, we did not evaluate certain clinical data that would have been worth analysing in older male patients with FUTI including the functional status, the presence of urinary incontinence, altered mental status or delirium. Thirdly, we excluded from the analysis FUTI episodes with negative or polymicrobial urine culture which might have induced a selection bias. Finally, the data collection was limited to the hospitalization period which might have underestimated the outcomes.

## Conclusions

Despite these limitations, our study indicates that older males with FUTI present different clinical characteristics and show specific microbiological features and increased antimicrobial resistance rates. In the multivariate analysis age was not associated with infections caused by MDRO.

## Data Availability

The datasets used and/or analysed during the current study are available from the corresponding author on reasonable request.

## Abbreviations

ABP: Acute bacterial prostatitis
ASB: Asymptomatic bacteriuria
BPH: Benign prostatic hyperplasia
ED: Emergency department
ESBL: Extended-spectrum betalactamase
FQ: Fluoroquinolones
FUTI: Febrile urinary tract infections
HCA: Healthcare-associated
IEAT: Inadequate empirical antimicrobial treatment
IUC: Indwelling urinary catheter
LTCF: Long-term care facility
LUTS: lower urinary tract symptoms
MAP: Mean arterial pressure
MDRO: Multidrug-resistant organisms
OPAT: Outpatient parenteral antimicrobial treatment
TGC: Third-generation cephalosporins
UTI: Urinary tract infections
